# Supplementary material for: Deep-coverage spatiotemporal proteome of the picoeukaryote Ostreococcus tauri reveals differential effects of environmental and endogenous 24-hour rhythms
Source: Commun Biol. 2021 Sep 30;4:1147. doi: 10.1038/s42003-021-02680-3 (PMC8484446; doi:10.1038/s42003-021-02680-3)
Supplement: Supplementary file 2 — Supplementary Information [file 42003_2021_2680_MOESM2_ESM.pdf]

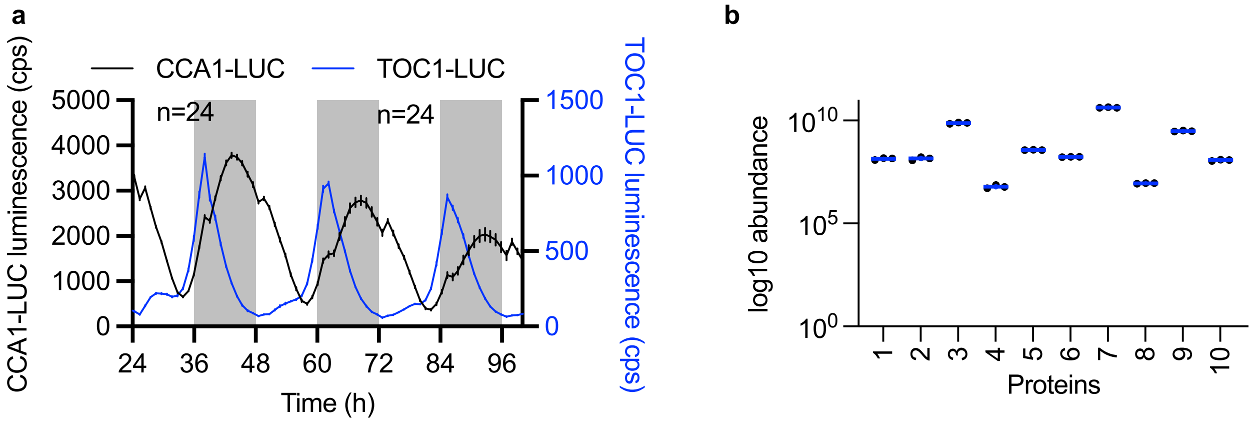


*Supplementary Figure 1*: **a** Luminescent traces of CCA1-LUC and TOC1-LUC in LD, at a ~1-h sampling rate (mean with SEM) showing that clock-regulated gene expression is qualitatively identical over different 24h cycles under entrainment. **b** Abundance data from 10 proteins, sampled randomly from the proteome results of a pilot experiment with three biological replicates (black data points). The mean values (blue line, with SEM) of the three replicates are provided to show the overlap with all three replicates. Based on these results the decision was taken to sample biological triplicates for the full time series but to pool these before proteomic analysis: the error bars cannot be distinguished and did not warrant the tripling of costs, processing time, and facility running times that would be incurred by running replicates separately. The main cause for this high reproducibility is probably that *Ostreococcus* is a highly uniform clonal cell line that is neither adherent nor actively motile. This is a very different type of biological sample than for example a set of Arabidopsis plants where two plants are never identical, or a number of mouse livers that have to be surgically removed from different animals, or a collection of Drosophila heads that are separated from their bodies by vortexing a heterogenous group of whole animals. The level of processing required for *Ostreococcus* is limited: we spin down identical volumes of three identical cultures, and freeze the pellets before protein extraction. Therefore, variation between biological material and extract preparation do not lead to a quantifiable error in this cell type.

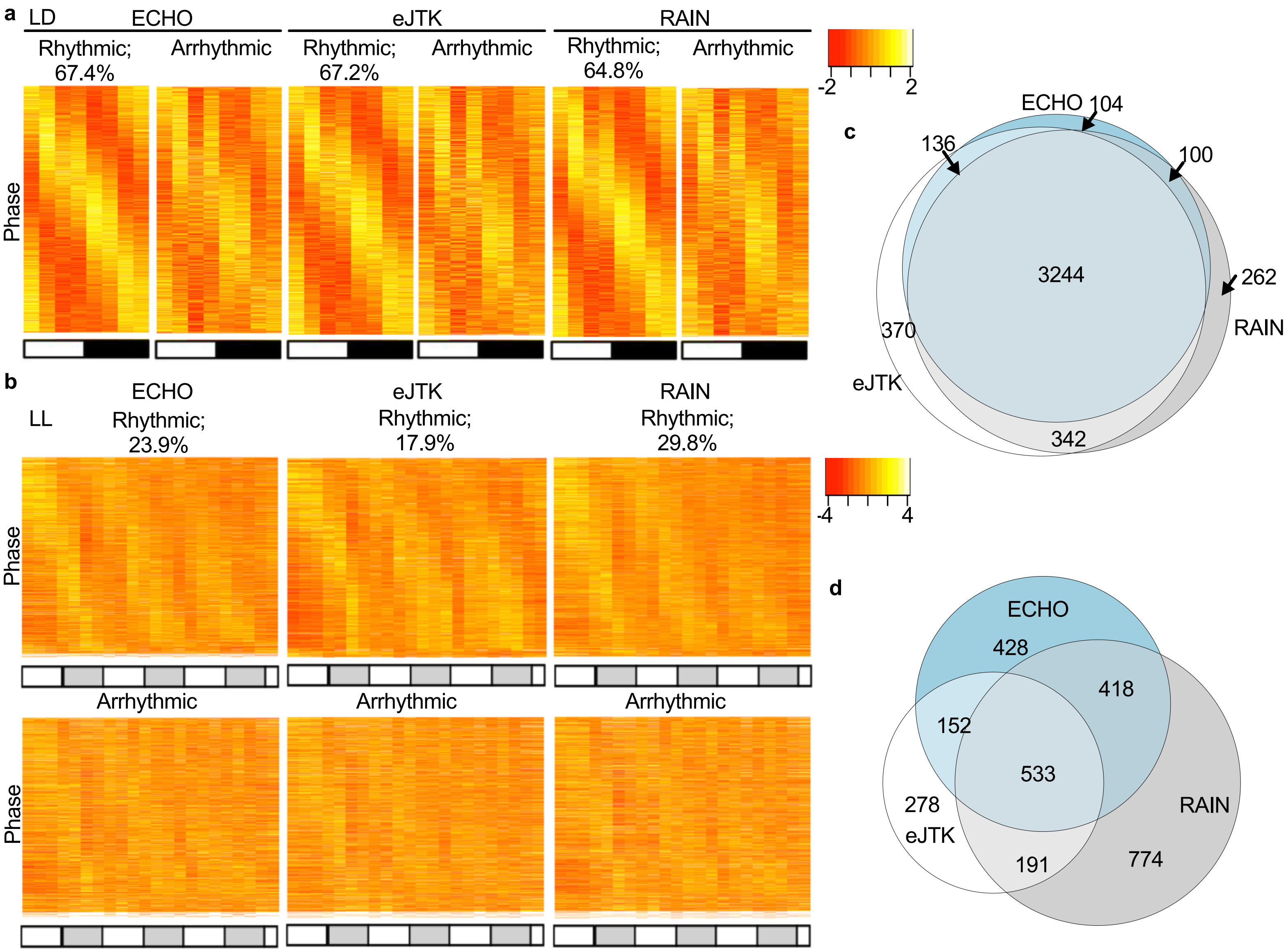


*Supplementary Figure 2: Algorithm choice for rhythmicity analyses*

**a-b** Heat maps showing min-max (red-yellow) normalised plots for all rhythmic proteins in LD (**a**) or LL (**b**) as determined using ECHO, eJTK or RAIN algorithms, where proteins with $p<0.05$ are considered rhythmic. Rows represent individual proteins, ordered by phase. Each column is a separate time point. Legends are Z-scores. **c-d** Venn diagrams showing the overlap in rhythmic proteins determined by each algorithm for LD (**c**) or LL (**d**). Source data can be found in Supplementary Data 2.

*Supplementary Figure 3*

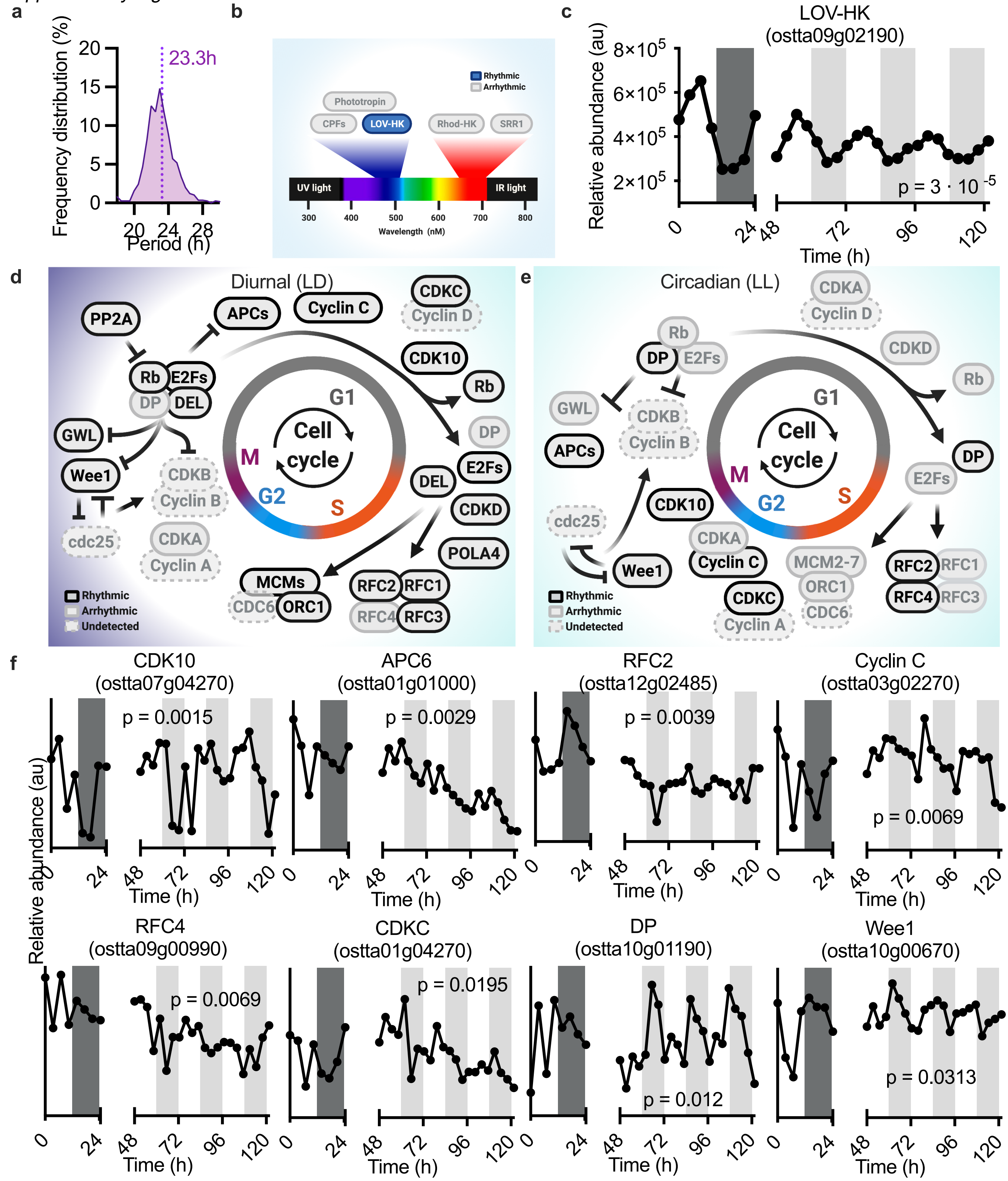


*For Legend, PTO*

*Supplementary Figure 3: Rhythmicity within the circadian clock and cell cycle*
**a** A frequency histogram of the period of oscillations observed under LL shows the expected mean value of ~23h. **b** Photoreceptors in the *Ostreococcus* proteome. *Ostreococcus* senses light changes in the environment through different blue and red light photoreceptors. The histidine kinase LOV-HK, Cryptochrome/Photolyase family (CPF) proteins, and Phototropin display UV/blue-light sensing properties, while red light fluencies are perceived by the histidine kinase Rhod-HK and SRR1[1]. We observed full coverage of all main photoreceptors, and remarkably rhythmic protein abundance is only evident for LOV-HK, under both entrained and free-running conditions. In *Ostreococcus*, LOV-HK is transcriptionally regulated by the clock around dawn and is also essential for synchronizing core clock components[2], not only upon blue light exposure but also in response to other light wavelengths, indicating interconnected light pathways. Interestingly, vertical distributions of *Ostreococcus* populations in aquatic environments suggest that UV/blue light is the main light source due to the wide blue light distribution at depth, which would help cells to discriminate time of day[2, 3, 4]. **c** Traces of quantified data from LD and LL time series for the LOV-HK protein. P value refers to the eJTK score in LL. **d-e** Reconstruction of the *Ostreococcus* cell cycle under LD (D) or LL (E), indicating the instructive rhythmic proteins. Our bioinformatic analyses identified 44 core cell cycle candidate genes in the *Ostreococcus* genome, of which we detected 35 of the protein products. Only 8 of these proteins were rhythmically abundant under LL and 22 proteins under LD. Interestingly, our analyses provide a detailed perspective on the tight control of cell cycle phases by the circadian clock under both conditions, with near-identical cell cycle stages. G1 proteins such as the CDKD or E2F factors as DEL only displayed rhythmicity under LD, suggesting no direct role of the circadian clock modulating G1 phase protein abundance. Specific subunits of the S phase RFC complex and the Pre-replication complex were rhythmically abundant under LD (RFCs, MCMs and ORC1) but only the RFC complex under LL, indicating that coupling of the circadian clock to the progression through S phase is mainly mediated through the RFC complex. Although several studies show no discrete activity of Cyclin C at any specific cell cycle phase[5], we found that Cyclin C protein abundance under LL rhythmically peaks at CT15, matching with the G2 phase in which cells prepare for cell division. This result would correlate with the major role in RNA transcription initiation of Cyclin C and the key transcription event of M phase-related genes before cell division observed in humans[6]. While rhythmic protein abundance of the key kinase Wee1 peaked at a similar phase under both conditions, other kinases such as CDK10 and CDKC displayed almost opposite rhythmic patterns under LL and LD. Wee1 is clock-regulated in other organisms and determines entry to M phase, and is also involved in other processes such as the DNA damage response[7, 8, 9, 10, 11]. Similarly, CDK10 and CDKC play important roles in genome stability, DNA repair and stress responses. These results could indicate a circadian contribution to preventing genome instability and maintaining DNA integrity, by regulating the G2/M transition under LL through the protein levels of Wee1, and a more flexible regulation of CDK10 and CDKC based on the light/dark cycles. During the M phase, although several proteins from the Retinoblastoma complex (Rb and E2Fs as DEL) and phosphatases as PP2A showed clear rhythmicity under LD, DP protein was the only rhythmic protein under LL peaking in the middle of the night. DP protein is an essential component of the Retinoblastoma complex, which is assembled at the end of the M phase to start a new cycle by inhibiting M phase-related proteins such as the rhythmic APC6-CDC16 from the anaphase-promoting complex (APC)[12, 13, 14]. This suggests a direct circadian regulation of the M phase through DP protein and additional light/dark regulation of other M phase proteins. **f** Traces of quantified data from LD and LL time series for examples of cell cycle proteins significantly rhythmic under LL. P values refer to eJTK scores under LL. Source data can be found in Supplementary Data 1.

*Supplementary Figure 4*

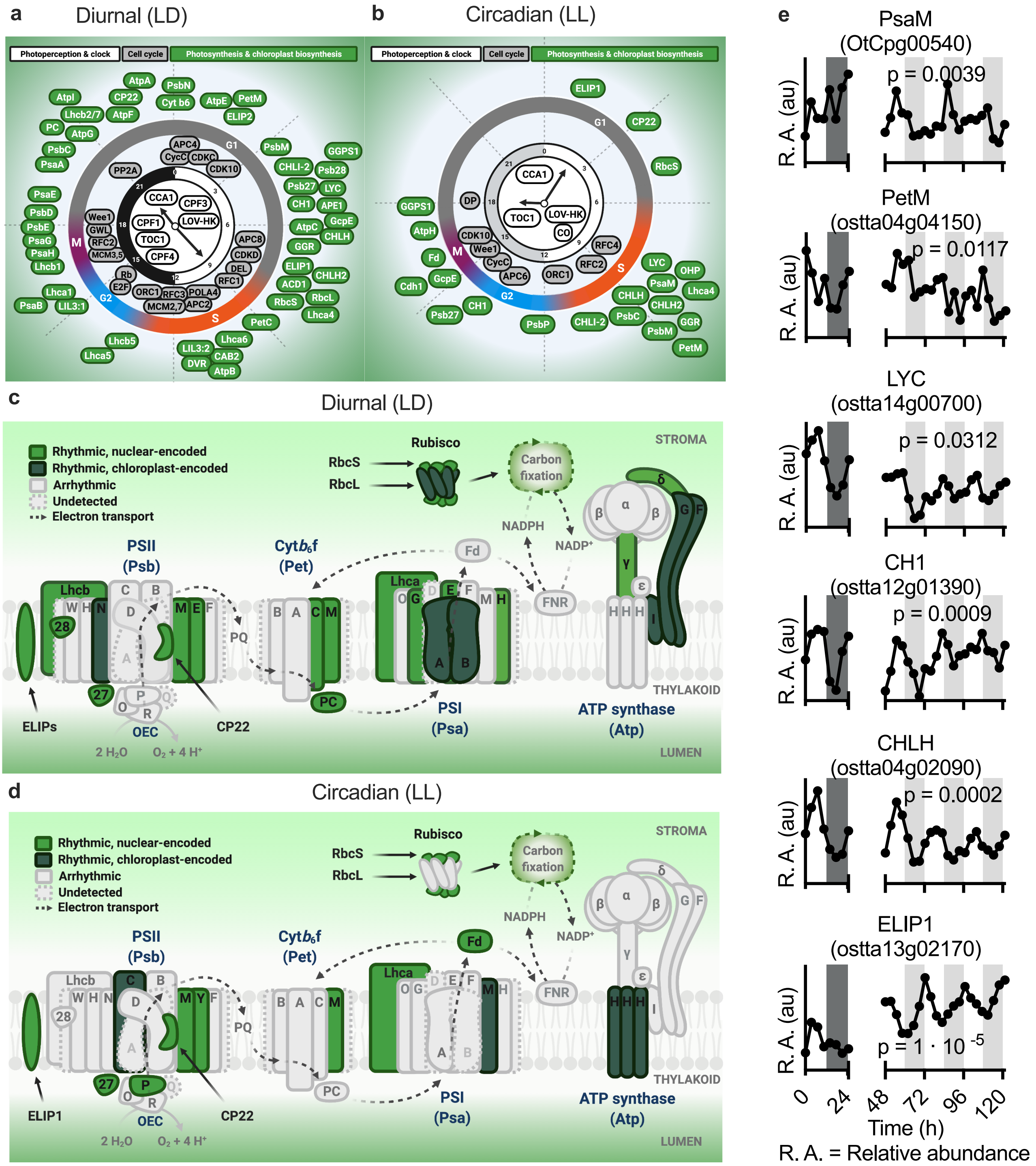


*For legends, PTO*

*Supplementary Figure 4: The rhythmically regulated proteins of the photosynthetic machinery*

**a-b** Diagrams related to Fig. 2A, but expanded to include output rhythms in photosynthetic and chloroplast biogenesis-associated proteins under LD (**a**) or LL (**b**). **c-d** Rhythmicity within the photosynthetic apparatus under LD (**c**) or LL (**d**) conditions. Circadian regulation of photosynthetic genes has been well described[15, 16, 17]. This apparatus consists of 4 main protein complexes, conserved from cyanobacteria to higher plants: Photosystem II (PSII), Cytochrome $b_6f$ (Cyt$b_6f$), Photosystem I (PSI), and ATP synthase. Among the 74 photosynthesis-related proteins we detected, a remarkably different organization of rhythmic proteins under LL compared to LD cycles was observed. All main photosynthetic complexes contain rhythmically abundant subunits. Specifically under LL, proteins from the photosynthetic apparatus (Psb, Pet, Psa) and pigment biosynthesis (Lycopene lyase (LYC) and Magnesium chelatases (CHLH, CHLH2)) were predominantly most abundant around CT8, coinciding with the start of the S phase of the cell cycle. Our results therefore match previous observations in algae[18] that suggest the onset of chloroplast division at the start of the S phase, ahead of cell cytokinesis. Consistent with that result, proteins involved in the later stages of photosystem assembly and chloroplast formation[19, 20], such as Psb27, peak subsequently. Under entrained conditions, abundance rhythms are more prevalent among photosynthetic proteins including those of the light harvesting complexes (LHCAs, LHCBs, LIL3 proteins), and these show a broader phase distribution over the light period. This indicates an important light/dark regulation of a subset of photosynthetic proteins separate from the circadian control. Proteins involved in energy-related processes at the end of the photosynthetic apparatus also showed differential rhythmicity under both conditions: subunits of the ATP synthase complex (Atps) were rhythmic under LD, but only the transmembrane ATPase subunit C (AtpH) and Ferredoxin peaked at night. Proteins involved in isoprenoid biosynthesis, such as Chlorophyll a oxygenase (CH1) and GeranylGeranyl Pyrophosphate Synthase 1 (GGPS1), peaked at opposite phases in LL compared to LD, which could correspond with known light-dark-dependent effects of downstream steps of pigment biosynthesis pathways[21, 22, 23]. Our results also showed rhythmic photoprotection-related proteins such as Early Light-Induced Proteins (ELIPs), CP22 or One Helix Protein (OHP) peaking during the day, which correlates with anticipation of photodamage by light exposure[24, 25]. Finally, protein abundance of the two subunits of the enzyme Rubisco (small subunit RbcS and large subunit RbcL) peaked at similar phases under LD, but only RbcS under LL suggesting circadian regulation of the enzyme Rubisco through RbcS[26]. Overall, proteomics results integrate well with previous studies from the clock, cell cycle, and light signalling fields, and illustrate in detail how pervasive rhythmicity throughout cellular biochemistry is achieved by circadian rhythms in the abundance of specific components within pathways. **e** Traces of quantified data from LD and LL time series for some examples of photosynthesis-related proteins significantly rhythmic under LL. P values refer to eJTK scores under LL. Source data can be found in Supplementary Data 1.

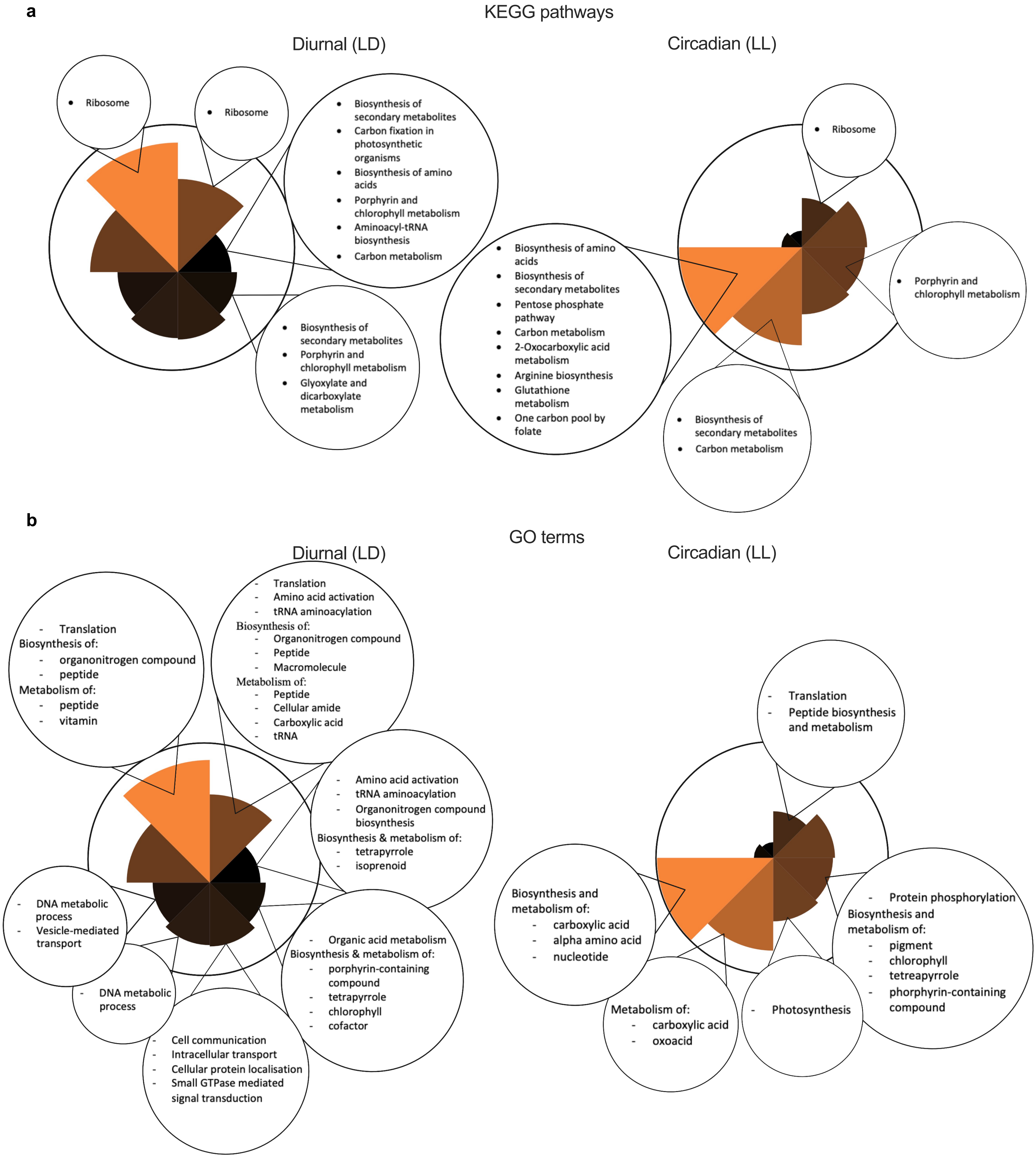


*Supplementary Figure 5: Enrichment of functional classifiers among protein peak phases*
**a** KEGG processes that are significantly enriched amongst rhythmic proteins in 3-hour peak phase windows in LD and LL.
**b** Biological process GO terms significantly enriched amongst rhythmic proteins in 3-hour peak phase windows in LD and LL.

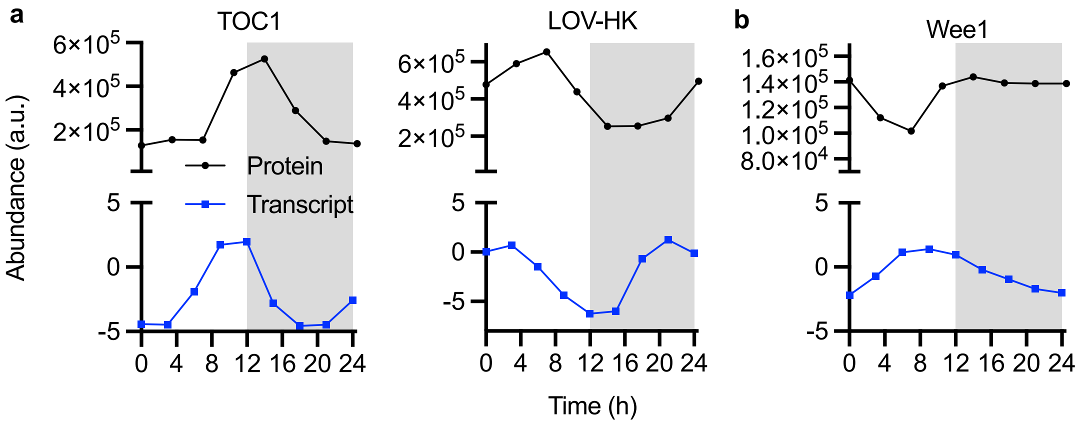


*Supplementary Figure 6: Relationships between transcript and protein phase under LD cycles*
**a** The transcript and protein profiles of the clock protein TOC1 and the photoreceptor LOV-HK show clear phase harmonics, indicating a linear relationship between protein abundance and the preceding mRNA abundance profile. **b** The transcript and protein profiles of the critical cell cycle kinase Wee1 are close to antiphasic, indicating a lack of phase harmonics. Source data can be found in Supplementary Data 1.

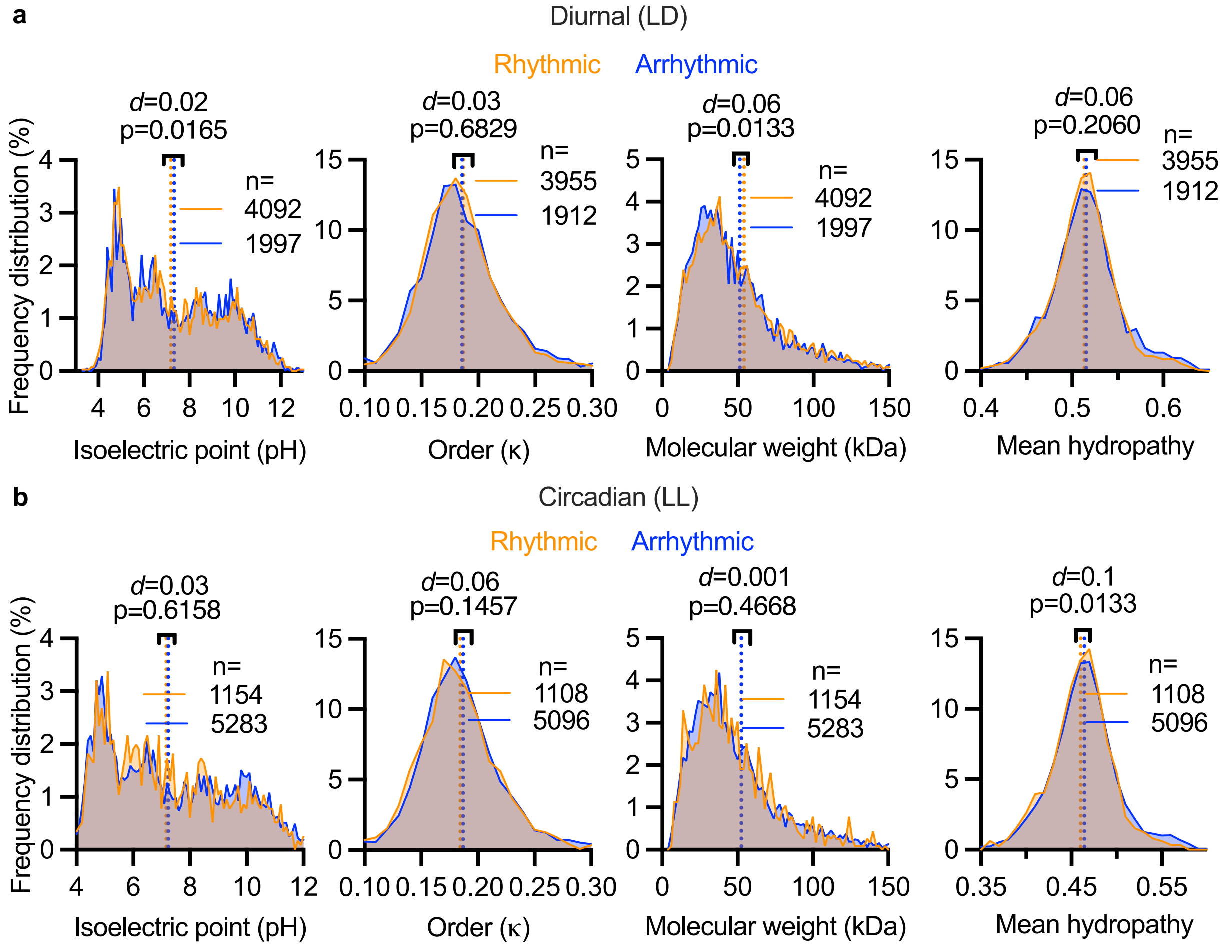


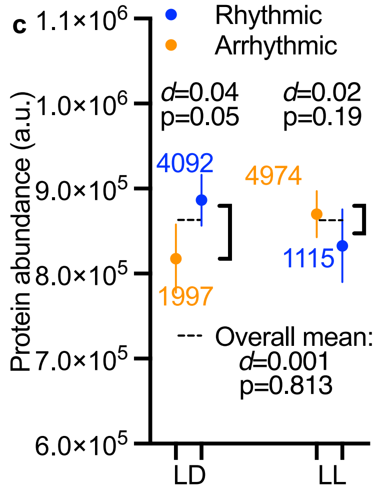


*Supplementary Figure 7: Biochemical properties of rhythmic versus arrhythmic proteins*

**a-b** The biochemical properties of rhythmic (orange) versus arrhythmic (blue) proteins under entrained (**a**) or constant (**b**) conditions. Differences are not statistically significant (Mann-Whitney tests p>0.01, and effect sizes are small (Cohen's *d* <0.2). **c** Mean protein abundance under LD and LL is the same for rhythmic (blue, mean with SEM) and arrhythmic proteins (orange). Dashed lines indicate the mean abundance across all proteins under each condition. Number of proteins in each of the four groups are indicated. Statistics (Mann-Whitney tests and Cohen's *d*) are provided to compare overall mean of all proteins as well as between rhythmic versus arrhythmic proteins. Source data can be found in Supplementary Data 1.

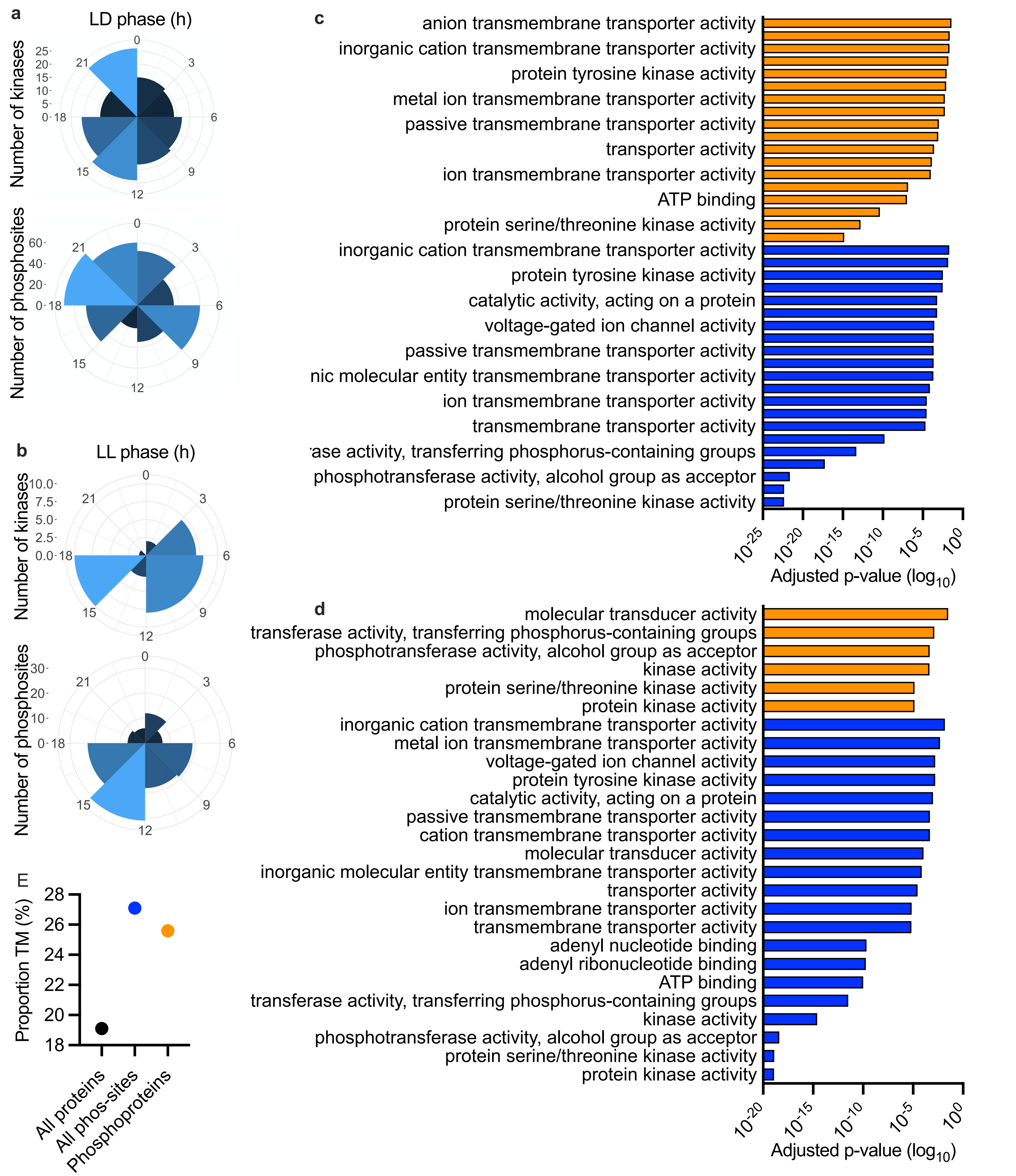


*Supplementary Figure 8: Rhythmic kinome and phosphoproteome regulate the transmembrane proteome*

**a-b** Circular histograms showing the number of rhythmic kinases (top) or phosphosites (bottom) in LD (**a**) or LL (**b**) at each 3-hour peak phase interval. No phospho-enrichment was performed; these data represent phosphopeptides detected in the original TMT runs. **c-d** Gene Ontology enrichment analysis for molecular function of all phosphorylated proteins (blue) or rhythmically phosphorylated proteins (orange) under LD (**c**) or LL (**d**). Under both conditions, membrane-associated functions (i.e. transporter activity) are overrepresented not only in phosphoproteins but also in rhythmically regulated phosphoproteins. **e** The percentage of transmembrane (TM) proteins among proteins carrying detected phosphorylation sites (blue) and among unique phosphoproteins (orange) is higher than among the overall detected proteome (black). Source data (except **c-d**) can be found in Supplementary Data 1.

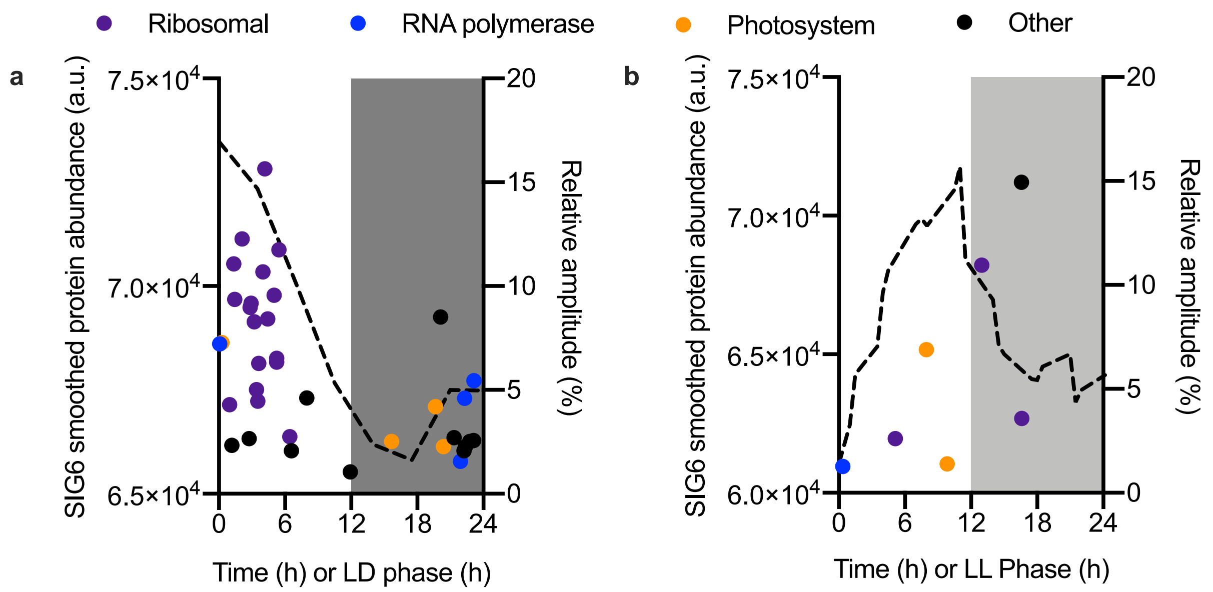


*Supplementary Figure 9: Phase relationship between the nuclear-encoded plastid transcription initiation factor SIG6 and the chloroplast-encoded proteome*

**a-b** Smoothed line of the mean abundance of SIG6 (left y-axes) under LD (**a**) or LL conditions (**b**) in relation to the amplitude and peak phase (data points; right y-axes) of all rhythmic chloroplast-encoded proteins. Purple dots represent ribosomal proteins, blue dots represent RNA polymerases, and orange dots represent photosystem components. Source data can be found in Supplementary Data 1

**Supplementary references**


1. Thommen Q, Pfeuty B, Schatt P, Bijoux A, Bouget FY, Lefranc M. Probing entrainment of Ostreococcus tauri circadian clock by green and blue light through a mathematical modeling approach. *Front Genet* **6**, 65 (2015).

2. Djouani-Tahri el B, Christie JM, Sanchez-Ferandin S, Sanchez F, Bouget FY, Corellou F. A eukaryotic LOV-histidine kinase with circadian clock function in the picoalga Ostreococcus. *Plant J* **65**, 578-588 (2011).

3. Chiang JY, Chen YC. Underwater image enhancement by wavelength compensation and dehazing. *IEEE Trans Image Process* **21**, 1756-1769 (2012).

4. Countway PD, Caron DA. Abundance and distribution of Ostreococcus sp. in the San Pedro Channel, California, as revealed by quantitative PCR. *Appl Environ Microbiol* **72**, 2496-2506 (2006).

5. Jezek J*, et al.* Cyclin C: The Story of a Non-Cycling Cyclin. *Biology (Basel)* **8**,  (2019).

6. Chaudhry MA, Chodosh LA, McKenna WG, Muschel RJ. Gene expression profiling of HeLa cells in G1 or G2 phases. *Oncogene* **21**, 1934-1942 (2002).

7. Matsuo T, Yamaguchi S, Mitsui S, Emi A, Shimoda F, Okamura H. Control mechanism of the circadian clock for timing of cell division in vivo. *Science* **302**, 255-259 (2003).

8. Gerard C, Goldbeter A. Entrainment of the mammalian cell cycle by the circadian clock: modeling two coupled cellular rhythms. *PLoS Comput Biol* **8**, e1002516 (2012).

9. Do K, Doroshow JH, Kummar S. Wee1 kinase as a target for cancer therapy. *Cell Cycle* **12**, 3159-3164 (2013).

10. Yu JH*, et al.* CDK10 functions as a tumor suppressor gene and regulates survivability of biliary tract cancer cells. *Oncol Rep* **27**, 1266-1276 (2012).

11. Guen VJ, Gamble C, Lees JA, Colas P. The awakening of the CDK10/Cyclin M protein kinase. *Oncotarget* **8**, 50174-50186 (2017).

12. Ramanujan A, Tiwari S. APC/C and retinoblastoma interaction: cross-talk of retinoblastoma protein with the ubiquitin proteasome pathway. *Biosci Rep* **36**, (2016).

13. Giacinti C, Giordano A. RB and cell cycle progression. *Oncogene* **25**, 5220-5227 (2006).

14. Magae J, Wu CL, Illenye S, Harlow E, Heintz NH. Nuclear localization of DP and E2F transcription factors by heterodimeric partners and retinoblastoma protein family members. *J Cell Sci* **109 ( Pt 7)**, 1717-1726 (1996).

15. Dodd AN, Belbin FE, Frank A, Webb AA. Interactions between circadian clocks and photosynthesis for the temporal and spatial coordination of metabolism. *Frontiers in plant science* **6**, 245 (2015).

16. Rust MJ, Golden SS, O'Shea EK. Light-driven changes in energy metabolism directly entrain the cyanobacterial circadian oscillator. *Science* **331**, 220-223 (2011).

17. Noordally ZB*, et al.* Circadian control of chloroplast transcription by a nuclear-encoded timing signal. *Science* **339**, 1316-1319 (2013).

18. Yoshida Y, Miyagishima SY, Kuroiwa H, Kuroiwa T. The plastid-dividing machinery: formation, constriction and fission. *Curr Opin Plant Biol* **15**, 714-721 (2012).

19. Liu H, Huang RY, Chen J, Gross ML, Pakrasi HB. Psb27, a transiently associated protein, binds to the chlorophyll binding protein CP43 in photosystem II assembly intermediates. *Proc Natl Acad Sci U S A* **108**, 18536-18541 (2011).

20. Allen JF, de Paula WB, Puthiyaveetil S, Nield J. A structural phylogenetic map for chloroplast photosynthesis. *Trends in plant science* **16**, 645-655 (2011).

21. Okada K, Saito T, Nakagawa T, Kawamukai M, Kamiya Y. Five geranylgeranyl diphosphate synthases expressed in different organs are localized into three subcellular compartments in Arabidopsis. *Plant Physiol* **122**, 1045-1056 (2000).

22. Lee S, Kim JH, Yoo ES, Lee CH, Hirochika H, An G. Differential regulation of chlorophyll a oxygenase genes in rice. *Plant Mol Biol* **57**, 805-818 (2005).

23. Querol J, Campos N, Imperial S, Boronat A, Rodriguez-Concepcion M. Functional analysis of the Arabidopsis thaliana GCPE protein involved in plastid isoprenoid biosynthesis. *FEBS Lett* **514**, 343-346 (2002).

24. Zavafer A, Cheah MH, Hillier W, Chow WS, Takahashi S. Photodamage to the oxygen evolving complex of photosystem II by visible light. *Sci Rep* **5**, 16363 (2015).

25. Adamska I, Kruse E, Kloppstech K. Stable insertion of the early light-induced proteins into etioplast membranes requires chlorophyll a. *J Biol Chem* **276**, 8582-8587 (2001).

26. Vitlin Gruber A, Feiz L. Rubisco Assembly in the Chloroplast. *Front Mol Biosci* **5**, 24 (2018).
