# Supplementary material for: Deep-coverage spatiotemporal proteome of the picoeukaryote Ostreococcus tauri reveals differential effects of environmental and endogenous 24-hour rhythms
Source: Commun Biol. 2021 Sep 30;4:1147. doi: 10.1038/s42003-021-02680-3 (PMC8484446; doi:10.1038/s42003-021-02680-3)
Supplement: Supplementary file 3 — Description of Supplementary Files [file 42003_2021_2680_MOESM3_ESM.pdf]

Description of Additional Supplementary Files

File name: Supplementary Data 1

Description: *Proteomic quantification data, circadian parameterization, and genome information.* Excel file containing the proteomic results for nuclear-encoded as well as organellar-encoded genome, along with the circadian analyses of the results and genome information. The file also contains the re-analysis of previously published transcriptome data, and lists of cell cycle and photosynthesis-associated proteins. Full legend is provided on the first tab of the excel file.

File name: Supplementary Data 2

Description: *Rhythmicity analyses of proteomics data.* The proteomics data were analysed by three methods (ECHO, RAIN, and eJTK) to assess rhythmicity of proteins under LD and LL conditions. The file lists p-values for each protein.
