# Supplementary material for: Deep-coverage spatiotemporal proteome of the picoeukaryote Ostreococcus tauri reveals differential effects of environmental and endogenous 24-hour rhythms
Source: Commun Biol. 2021 Sep 30;4:1147. doi: 10.1038/s42003-021-02680-3 (PMC8484446; doi:10.1038/s42003-021-02680-3)
Supplement: Supplementary file 6 — Reporting Summary [file 42003_2021_2680_MOESM6_ESM.pdf]

nature research

Corresponding author(s): Gerben van Ooijen

Last updated by author(s): Aug 17, 2021

Reporting Summary

Nature Research wishes to improve the reproducibility of the work that we publish. This form provides structure for consistency and transparency in reporting. For further information on Nature Research policies, see our Editorial Policies and the Editorial Policy Checklist.

Statistics

For all statistical analyses, confirm that the following items are present in the figure legend, table legend, main text, or Methods section.

n/a	Confirmed	
☐	☒	The exact sample size (*n*) for each experimental group/condition, given as a discrete number and unit of measurement
☐	☒	A statement on whether measurements were taken from distinct samples or whether the same sample was measured repeatedly
☐	☒	The statistical test(s) used AND whether they are one- or two-sided<br>*Only common tests should be described solely by name; describe more complex techniques in the Methods section.*
☐	☒	A description of all covariates tested
☐	☒	A description of any assumptions or corrections, such as tests of normality and adjustment for multiple comparisons
☐	☒	A full description of the statistical parameters including central tendency (e.g. means) or other basic estimates (e.g. regression coefficient) AND variation (e.g. standard deviation) or associated estimates of uncertainty (e.g. confidence intervals)
☒	☐	For null hypothesis testing, the test statistic (e.g. *F*, *t*, *r*) with confidence intervals, effect sizes, degrees of freedom and *P* value noted<br>*Give P values as exact values whenever suitable.*
☒	☐	For Bayesian analysis, information on the choice of priors and Markov chain Monte Carlo settings
☒	☐	For hierarchical and complex designs, identification of the appropriate level for tests and full reporting of outcomes
☐	☒	Estimates of effect sizes (e.g. Cohen's *d*, Pearson's *r*), indicating how they were calculated

Our web collection on statistics for biologists contains articles on many of the points above.

Software and code

Policy information about availability of computer code

Data collection	*Provide a description of all commercial, open source and custom code used to collect the data in this study, specifying the version used OR state that no software was used.*
Data analysis	MaxQuant v 1.6.6.0<br>BioDare2<br>GraphPad Prism v9<br>R v3.6.1

For manuscripts utilizing custom algorithms or software that are central to the research but not yet described in published literature, software must be made available to editors and reviewers. We strongly encourage code deposition in a community repository (e.g. GitHub). See the Nature Research guidelines for submitting code & software for further information.

Data

Policy information about availability of data

All manuscripts must include a data availability statement. This statement should provide the following information, where applicable:

- Accession codes, unique identifiers, or web links for publicly available datasets
- A list of figures that have associated raw data
- A description of any restrictions on data availability

The mass spectrometry proteomics data have been deposited to the ProteomeXchange Consortium via the PRIDE74 partner repository with the dataset identifier PXD025009. Supplementary Data 1 contains the source data for all main figures except Figure 2b-c, and for Supplementary Figures 3, 4, 6, 7, 8a-b, and 9. Supplementary Data 2 contains source data for Supplementary Figure 2.

Field-specific reporting

Please select the one below that is the best fit for your research. If you are not sure, read the appropriate sections before making your selection.

[x] Life sciences   [ ] Behavioural & social sciences   [ ] Ecological, evolutionary & environmental sciences

For a reference copy of the document with all sections, see nature.com/documents/nr-reporting-summary-flat.pdf

Life sciences study design

All studies must disclose on these points even when the disclosure is negative.

Sample size	No sample size calculation was performed. For a large-scale proteomics time series, 3 replicates (each containing around 700 million algal cells) per time point were considered sufficient based on the preliminary experiment as reported in Supplementary Figure 1.
Data exclusions	No data were excluded.
Replication	For the proteomics time series, 3 replicates were collected per time point and these were pooled before mass spectrometric analysis. All other experiments are representative of at least two replicate experiments.
Randomization	Time series samples were randomised and relabelled before being processed.
Blinding	The mass spectrometry and processing were carried using randomised labels, with no knowledge of the original sequence of sample collection by the operator. Time points were unscrambled after normalisation of runs.

Reporting for specific materials, systems and methods

We require information from authors about some types of materials, experimental systems and methods used in many studies. Here, indicate whether each material, system or method listed is relevant to your study. If you are not sure if a list item applies to your research, read the appropriate section before selecting a response.

Materials & experimental systems

n/a	Involved in the study
[x]	[ ] Antibodies
[ ]	[x] Eukaryotic cell lines
[x]	[ ] Palaeontology and archaeology
[x]	[ ] Animals and other organisms
[x]	[ ] Human research participants
[x]	[ ] Clinical data
[x]	[ ] Dual use research of concern

Methods

n/a	Involved in the study
[x]	[ ] ChIP-seq
[x]	[ ] Flow cytometry
[x]	[ ] MRI-based neuroimaging

Eukaryotic cell lines

Policy information about cell lines

Cell line source(s)	All cell lines (WT, CCA1-LUC, and TOC1-LUC) are Ostreococcus tauri OTTH0595 (RCC745) and were obtained from the Bouget lab as published in Corellou F, Schwartz C, Motta JP, el Djouani-Tahri B, Sanchez F. , et al (2009) Clocks in the green lineage: comparative functional analysis of the circadian architecture of the picoeukaryote Ostreococcus. Plant Cell 21: 3436-3449.
Authentication	The luminescent cell lines were authenticated by circadian parameters of luminescence and by western blot of the transgene.
Mycoplasma contamination	Mycoplasma is not of relevance to this cell type or media. Therefore none of the lines were tested.
Commonly misidentified lines (See ICLAC register)	We refer to 'CCA1' for the gene ostta06g02340, as consistent with all but one previous study on this orgabnism. In a single unrelated study, ostta06g01220 is erroneously referred to as CCA1: De Los Reyes et al., Frontiers in Plant Science 2017.
